# The Bacterial Gut Microbiota of Adult Patients Infected, Colonized or Noncolonized by *Clostridioides difficile*

**DOI:** 10.3390/microorganisms8050677

**Published:** 2020-05-06

**Authors:** Monique J. T. Crobach, Quinten R. Ducarmon, Elisabeth M. Terveer, Celine Harmanus, Ingrid M. J. G. Sanders, Kees M. Verduin, Ed J. Kuijper, Romy D. Zwittink

**Affiliations:** 1 Experimental Bacteriology, Department of Medical Microbiology, Center for Infectious Diseases, Leiden University Medical Center, 2333ZA Leiden, The Netherlands; m.j.t.crobach@lumc.nl (M.J.T.C.); q.r.ducarmon@lumc.nl (Q.R.D.); e.m.terveer@lumc.nl (E.M.T.); c.harmanus@lumc.nl (C.H.); i.m.j.g.sanders@lumc.nl (I.M.J.G.S.); e.j.kuijper@lumc.nl (E.J.K.); 2 Center for Microbiome Analyses and Therapeutics, Department of Medical Microbiology, Center for Infectious Diseases, Leiden University Medical Center, 2333ZA Leiden, The Netherlands; 3 Netherlands Donor Feces Bank, 2333ZA Leiden, The Netherlands; 4 Department of Microbiology and Infection Prevention, Amphia Hospital, 4818CK Breda, The Netherlands; k.verduin@pamm.nl

**Keywords:** *Clostridioides difficile*, *Clostridium difficile*, gut microbiota, colonization, 16S rRNA gene amplicon sequencing

## Abstract

Gut microbiota composition in patients with *Clostridioides difficile* colonization is not well investigated. We aimed to identify bacterial signatures associated with resistance and susceptibility to *C. difficile* colonization (CDC) and infection (CDI). Therefore, gut microbiota composition from patients with CDC (*n* = 41), with CDI (*n* = 41), and without CDC (controls, *n* = 43) was determined through 16S rRNA gene amplicon sequencing. Bacterial diversity was decreased in CDC and CDI patients (*p* < 0.01). Overall microbiota composition was significantly different between control, CDC, and CDI patients (*p* = 0.001). Relative abundance of *Clostridioides* (most likely *C. difficile*) increased stepwise from controls to CDC and CDI patients. In addition, differential abundance analysis revealed that CDI patients’ gut microbiota was characterized by significantly higher relative abundance of *Bacteroides* and *Veillonella* than CDC patients and controls. Control patients had significantly higher *Eubacterium hallii* and *Fusicatenibacter* abundance than colonized patients. Network analysis indicated that *Fusicatenibacter* was negatively associated with *Clostridioides* in CDI patients, while *Veillonella* was positively associated with *Clostridioides* in CDC patients. Bacterial microbiota diversity decreased in both CDC and CDI patients, but harbored a distinct microbiota. *Eubacterium hallii* and *Fusicatenibacter* may indicate resistance against *C. difficile* colonization and subsequent infection, while *Veillonella* may indicate susceptibility to colonization and infection by *C. difficile*.

## 1. Introduction

*Clostridioides difficile*, formerly named *Clostridium difficile*, is an anaerobic, Gram-positive, spore-forming bacillus. It is the main causative agent of nosocomial diarrhea, with antibiotic use as its most important risk factor. Nowadays, community-associated diarrhea due to *C. difficile* is also increasingly reported. Clinical symptoms arise when *C. difficile* spores germinate within the intestine and the viable bacteria start to produce toxins. The secretion of Toxin A (TcdA) and Toxin B (TcdB) leads to inflammation of the large intestine [1]. The clinical presentation may range from mild diarrhea to a life-threatening toxic megacolon [1]. However, the ingestion of *C. difficile* spores does not always lead to the development of symptomatic disease. *C. difficile* can also be silently present in the gut, without causing any symptoms. This condition is called asymptomatic *C. difficile* colonization [2]. Patients colonized with *C. difficile* play an important role in disease epidemiology, as they act as a reservoir for onward transmissions [3,4]; they may also progress to infection themselves, especially in the presence of an underlying illness [5,6,7].

It is believed that the bacterial gut microbiota plays an important role in determining the susceptibility to colonization and subsequent infection with *C. difficile*. In patients with *C. difficile* infection (CDI), a lower richness and diversity, and decreased relative abundances of Bacteroidetes, *Ruminococcaceae* and *Lachnospiraceae* members have been described [8,9]. The gut microbiota in *C. difficile* colonized patients is less well characterized [8,10], but may give more insight into mechanisms that allow for colonization whilst protecting against infection. A previous study identified specific gut metabolites associated with colonization and infection by *C. difficile*, but did not determine microbiota composition [11]. In order to identify which bacterial signatures are associated with resistance and susceptibility to *C. difficile* colonization and CDI, we compared the gut microbiota of CDI patients, patients with *C. difficile* colonization (CDC) at hospital admission, and patients without CDI or CDC at admission.

## 2. Materials and Methods

### 2.1. Subjects and Sample Collection

This study was designed to compare the gut microbiota between three groups: patients with *C. difficile* colonization (CDC) on hospital admission, patients without *C. difficile* colonization on hospital admission (controls), and hospitalized patients with CDI. For the first two groups, fecal samples were obtained from CDC and control patients admitted to Leiden University Medical Center (LUMC) or Amphia hospital as part of the CDD (“*Clostridium difficile* dragerschap” (carriership)) study, a study designed to determine the prevalence of CDC at hospital admission, conducted between January 2015 and March 2016. Adult patients admitted to predefined medical and surgical wards were eligible for enrolment. Stool samples were requested within 72 h of hospital admission. If patients were discharged home within 72 h, a stool sample was collected at home and returned to the hospital by mail or in person. Colonized patients were defined as patients who tested positive for *C. difficile* by stool culture and were not clinically suspected of CDI within the first 72 h of admission. For each colonized patient, the first consecutive patient with a negative stool culture for *C. difficile* was included to form the control group. For the third group, fecal samples were obtained from adult patients hospitalized in the LUMC and diagnosed with CDI between July 2015 and May 2017. All CDI cases had to comply with the definitions valid in the Dutch surveillance protocol [12], and CDI diagnosis was based on CDI symptoms in combination with laboratory CDI testing in agreement with the recommendations of the European Centre for Disease Control and Prevention [13]. *C. difficile* culturing and molecular diagnostics were performed as described below in Section 2.2. Patients initially participating in the CDD study but diagnosed with CDI within 72 h of admission were included in the CDI group.

The LUMC institutional review board served as the central institutional review board and had no objection to the performance of the study. At the Amphia hospital, the directing board had no objection to the performance of the study. Stool samples from CDC and control patients were collected under verbal consent, and written informed consent from these patients was obtained for collection of additional data (see below). A waiver for informed consent from CDI patients was obtained.

### 2.2. Microbiological Analyses

Microbiological analyses were performed at the National Reference Laboratory for Clostridium difficile (LUMC, The Netherlands). Fecal samples were initially stored at 2–6 °C and tested on the day of receipt, or the following working day in case of weekends or holidays.

Fecal samples from CDC and control patients were cultured on CLO plates (containing cefoxitin, amphotericin B and cycloserin, BioMérieux, The Netherlands) and after ethanol shock on CLO plates and CNA plates (containing colistin and nalidixinic acid, BioMérieux, The Netherlands). Suspicious colonies were tested by GDH PCR to confirm the presence of *C. difficile* [14]. In addition, a multiplex PCR for TcdA, TcdB, and binary toxin genes was performed on the isolates to determine if CDC patients were colonized by a toxigenic or nontoxigenic strain [15].

Fecal samples from patients with suspected CDI were tested according to standard operating procedures, which included an assay to detect free *C. difficile* toxins [16]. In addition, positive tested samples were cultured for presence of *C. difficile* as described above.

*C. difficile* isolates from CDC patients and CDI patients were PCR ribotyped as previously described [17].

### 2.3. Patient Data Collection

Demographical data and data about medication use during the last three months (until admission for CDC patients and controls or until sample submission for CDI patients), previous hospitalization in the last year, and previous CDI episodes (ever and within the last eight weeks) were collected by questionnaires and electronic chart review (CDC and control patients) or chart review only (CDI patients). Recurrent CDI was defined as a new diarrheal episode within two to eight weeks after a previous diarrheal episode due to *C. difficile* and *C. difficile* reinfection as a new diarrheal episode more than 8 weeks after the previous diarrheal episode due to *C. difficile*.

Epidemiological analyses were performed to compare characteristics between control, CDC, and CDI patients by one-way ANOVA or chi-squared test using STATA SE version 15.1 (StataCorp, College Station, TX, USA).

### 2.4. Microbiota Analysis

#### 2.4.1. Samples

A total of 125 fecal samples were included: 43 samples from control patients, 41 samples from CDC patients, and 41 samples from CDI patients. Samples from control and CDC patients were in 74/84 patients (88%) obtained within 72 h after admission. Fecal samples were submitted from home by 15/84 patients (17.9%), while from the other 69/84 patients (82.1%) fecal samples were collected in the hospital.

#### 2.4.2. DNA Extraction, Library Preparation and Sequencing

DNA was extracted from 0.1 g feces using the Quick-DNA™ Fecal/Soil Microbe Miniprep Kit (ZymoResearch, CA, USA). Quality control, library preparation, and sequencing were performed by GenomeScan B.V. (Leiden, The Netherlands) using the NEXTflex™ 16S V4 Amplicon-Seq Kit (BiooScientific, TX, USA) and the Illumina HiSeq4000 platform (paired-end, 150bp). An average of 2,117,322 (707,362–5,742,717) reads per sample was obtained. Raw sequencing data are available in the European Nucleotide Archive (http://www.ebi.ac.uk/ena) under study accession PRJEB30586.

#### 2.4.3. Sequencing Data Analysis

Read filtering, operational taxonomic unit (OTU)-picking, and taxonomic assignment were performed using the NGTax 0.4 pipeline with following settings: forward read length of 150, reverse read length of 120, ratio OTU abundance of 2.0, classify ratio of 0.9, minimum threshold of 1 × 10^−7^, identity level of 97%, and error correction of 98.5, using the Silva_132_SSU Ref database [18,19,20]. The obtained OTU-table was filtered for OTUs with a number of sequences less than 0.005% of the total number of sequences [21]. A couple of technical duplicates were included for DNA extraction (*n* = 3 samples) and sequencing (*n* = 6 samples) procedures, indicating high replicability of results (Figure S1). Three negative controls were included from DNA extraction onwards and contained less than 1% of the number of reads obtained from fecal samples.

All microbiota analyses and data visualization were performed in R (v3.5.1), using the packages phyloseq (v1.24.2), vegan (v2.5-2), ggplot2 (v3.0.0), DESeq2 (v1.20.0), microbiome (v1.2.1), and SpiecEasi (v0.1.4) [22,23,24,25,26,27]. Visualization of network analysis was performed in Cytoscape (v3.7.0) [28]. Results were considered significant if *p* ≤ 0.05, or Benjamini-Hochberg corrected *p* ≤ 0.05 for differential abundance analysis. Prior to differential abundance testing (DESeq2) and network analysis (SpiecEasi), the OTU-table was filtered for OTUs present in less than 25% of samples. Nucleotide sequences belonging to the *Clostridioides* genus were blasted using the NCBI standard nucleotide blast, with 16S ribosomal sequences (Bacteria and Archaea) selected as the reference database, to determine if the sequence had a better hit to *C. difficile* or *C. mangenotii*. Kruskal–Wallis followed by post-hoc Dunn’s testing was performed to compare Shannon diversity indices between the patient groups. Permutational multivariate analysis of variance (PERMANOVA) was performed using the “adonis” function with 999 permutations and Bray–Curtis distances to separately investigate associations between microbiota composition and various clinical variables. Each clinical variable was tested separately using PERMANOVA. SpiecEasi, using the Meinshausen–Buhlman method for graph estimation of the network, was performed for network analysis with lambda.min.ratio = 0.01, nlambda = 20 and rep.num = 99. This method is robust to many characteristics of 16S amplicon data, such as compositionality and dimensionality [22]. OTUs without a direct edge connection to another OTU were removed for visualization purposes.

## 3. Results

### 3.1. Epidemiology

Thirty CDI episodes were primary episodes, seven were recurrent episodes, and four were *C. difficile* reinfections. From four patients, two different episodes were included in this study. This mixture of primary episodes, recurrences, and reinfections reflects the true CDI population, as recurrence and reinfection are common. Previous CDI, both within and beyond the last eight weeks, was common among CDI patients (17.1% for both), whereas it was uncommon in CDC patients (2.4% and 7.3%), and no previous CDI was recorded in controls. Antibiotics were used in the last three months in 97.6% of CDI patients, 73.2% of CDC patients, and 59.5% of control patients (*p* < 0.001). The *C. difficile* PCR ribotype distribution in CDC and CDI patients is shown in Figure S2. Patient characteristics are shown in Table 1.

### 3.2. Bacterial Community Structure

**Figure 1:**
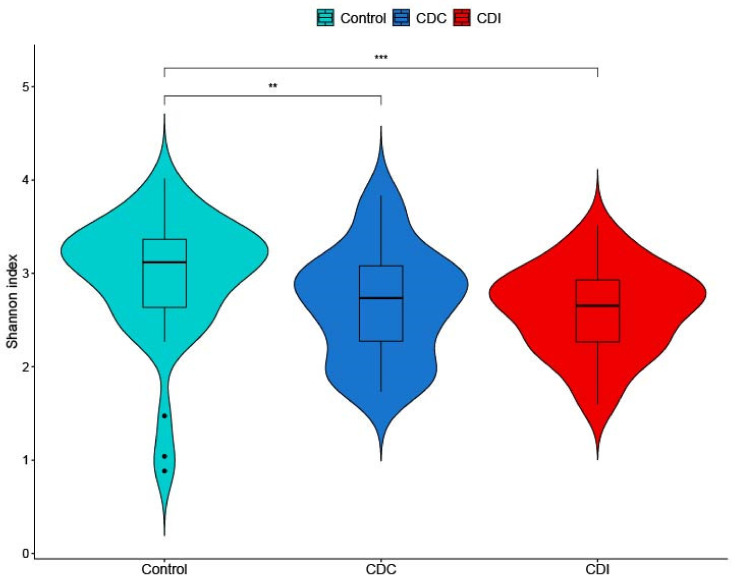
Violin plot of alpha diversity, as measured by the Shannon index, in control, *C. difficile* colonization (CDC) and *C. difficile* infection (CDI) patients. The box plot shows the median, 25th, and 75th percentiles, and whiskers indicate 1.5* interquartile range. * *p* < 0.05, ** *p* < 0.01, and *** *p* < 0.001.

To elucidate characteristics of the bacterial community structure, several tests were performed. We determined bacterial diversity using the Shannon index, performed PERMANOVA to relate microbiota composition to clinical factors, and clustered samples based on both weighted and unweighted UniFrac distance metrices for between-sample comparisons. Bacterial diversity was significantly higher in controls than in CDC and CDI patients (*p* < 0.01), but did not differ between CDC and CDI patients (Figure 1).

**Figure 2:**
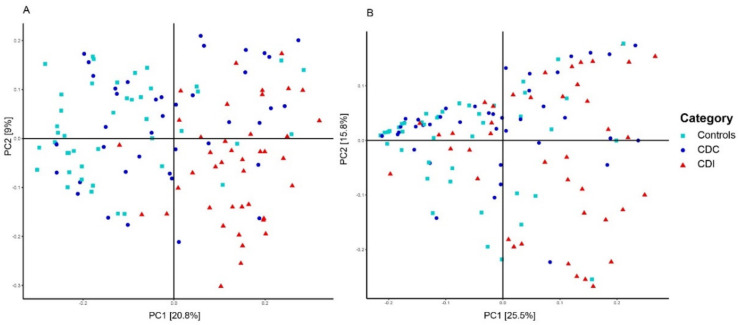
Principal Coordinates Analysis (PCoA) based on unweighted (**A**) and weighted (**B**) UniFrac distances. Each sample is represented by a shape and colur according to its category. The percentage of variation explained by the two first PCoA dimensions is indicated on the respective axes.

The most important clinical factor associated with microbiota composition was the patient group (PERMANOVA, *p* = 0.001, R^2^ = 0.075). Additional pairwise comparisons revealed that microbiota composition of control, CDC, and CDI patients all differed from each other (PERMANOVA, *p* < 0.01). The difference in microbiota composition between groups could also be observed via sample clustering based on unweighted UniFrac distance, but was less apparent, although still visible, using weighted UniFrac distance (Figure 2), reflecting differences in presence/absence of bacterial taxa rather than in their relative abundance. Here, microbiota composition of CDC patients are scattered, with some samples being more similar to CDI patients and others to controls. Clustering analysis solely on the CDC group showed no differentiation in microbiota composition by toxinogenic or non-toxinogenic *C. difficile* carriership, or by any other variable (data not shown).

In addition to patient group, overall microbiota composition was significantly affected by solid organ transplantation, previous CDI, PPIs/antacids, immunosuppressants, and specific antibiotics, including vancomycin and metronidazole, which are commonly prescribed antibiotics for CDI treatment (Table S1). However, effect sizes were smaller for these clinical variables than for segregation by patient group. Since antibiotics are known to alter gut microbiota composition, we explored whether antibiotic use in the previous three months affected microbiota composition within the control and CDC group. This was indeed the case for control patients (PERMANOVA, *p* = 0.035, R^2^ = 0.044), but not for CDC patients (PERMANOVA, *p* = 0.409, R^2^ = 0.031). Within the control group, antibiotic use also impacted bacterial diversity, with a trend for increased diversity in the nonantibiotic group (*p* = 0.0518). For these reasons, the control group was separated in controls with (C+AB) and without (C-AB) previous antibiotic use for differential abundance analysis.

### 3.3. Relative Abundance of Individual Bacterial Taxa

In order to study the differential abundance of bacterial taxa between the patient groups, DESeq2 analysis was performed. Relative abundance of *Clostridioides* showed a significant stepwise increase from C-AB (<0.01 ± <0.01%), C+AB (0.05 ± 0.2%) to CDC (0.7 ± 2.2%), and CDI patients (2.5 ± 2.9%) (Table S2A,B). It is, however, important to take prevalence into consideration, as *Clostridioides* reads were detected in only 26/41 CDC patients (63.4%) and in 38/41 CDI patients (92.7%). The nucleotide sequence belonging to this *Clostridioides* OTU resulted in a 100% sequence identity with two *C. difficile* strains, but only a 94% sequence identity with *C. mangenotii*.

**Figure 3:**
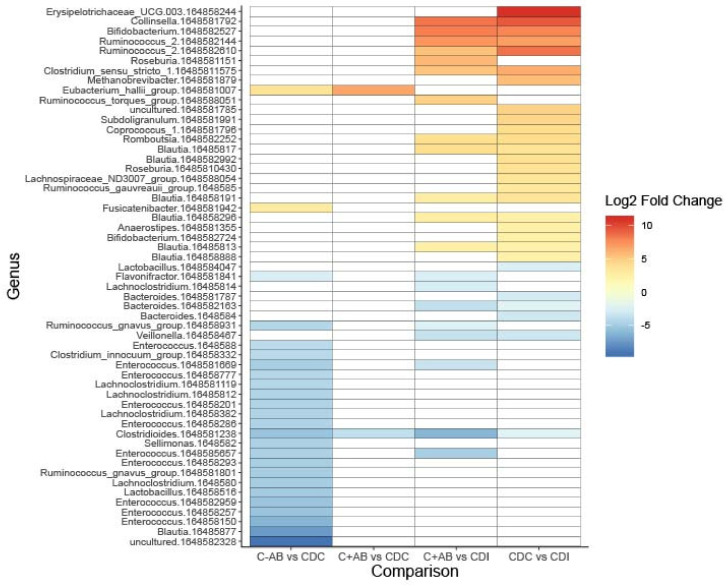
Heatmap showing differentially abundant bacterial taxa between without previous antibiotic use (C-AB), with previous antibiotic use (C+AB), CDC and CDI patients. Bacterial taxa with a Log2 fold change of at least (-)2.25 and a Benjamini–Hochberg corrected *p*-value ≤ 0.05 are shown on operational taxonomic unit (OTU)-level. OTU numbers are indicated as 164858xxxxxx. A full overview can be found in Table S2A.

Compared to CDC patients, C+AB and C-AB had an increased relative abundance of *Eubacterium hallii* (Figure 3, Table S2A,B). As expected, more and larger differences were observed between C-AB and CDC patients than between C+AB and CDC patients. In addition to an increase in *E. hallii,* the relative abundance of *Fusicatenibacter* was significantly higher in C-AB than in CDC patients, while the relative abundance of several *Enterococci*, *Ruminococcus gnavus,* and *Lachnoclostridium* were significantly lower (Figure 3, Table S2A,B).

Compared to C+AB and CDC patients, microbiota of CDI patients was characterized by a higher relative abundance of *Clostridioides*, *Bacteroides,* and *Veillonella*, and by a lower abundance of genera belonging to the *Ruminococcaceae* family and Actinobacteria phylum (Figure 3, Table S2A,B). Many of these lower abundant genera are known short-chain fatty acids (SCFA)-producers and carbohydrate degraders. Additionally, CDI patients had increased relative abundance of *R. gnavus* and *Lachnoclostridium* compared to C+AB patients. To avoid antibiotic use bias, CDI patients were not compared to C-AB patients.

### 3.4. Bacterial Networks

**Figure 4:**
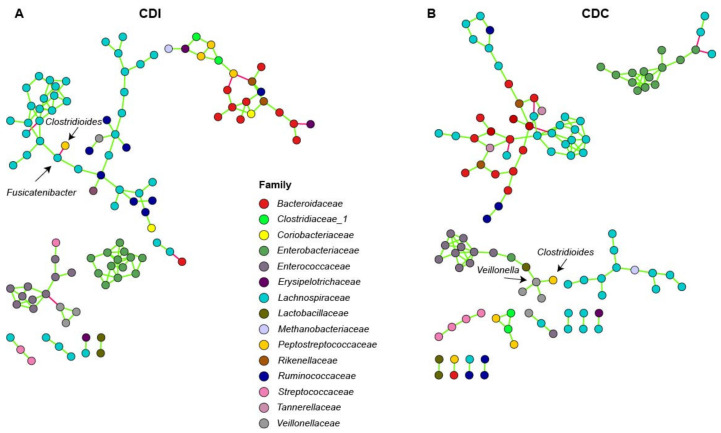
Association network analysis using SpiecEasi in CDI (**A**) and CDC (**B**) patients. Each node represents a single OTU and is colored according to family-level taxonomy. Green edges indicates a positive association between nodes, and red edges indicate a negative association between nodes.

To investigate connectivity of the differentially abundant *Clostridioides* genus with other bacterial genera, network analysis was performed on microbiota composition profiles of CDC and CDI patients. In CDI patients, *Fusicatenibacter* was negatively associated with *Clostridioides* (Figure 4A). In CDC patients, a positive association between *Clostridioides* and *Veillonella* was observed (Figure 4B). 

## 4. Discussion

It is generally accepted that CDI can develop due to a disturbed gut microbiota. In contrast, not much is known about the role of the gut microbiota in *C. difficile* colonization. In this study, the gut microbiota of patients with asymptomatic *C. difficile* colonization was characterized. CDC patients had unique gut microbiota signatures, and bacterial taxa could be identified that may be of relevance for further mechanistic studies. While 16S rRNA gene amplicon sequencing did not allow for identification of *Clostridioides* in all colonized and CDI patients, its relative abundance increased in a step-wise manner from controls to colonized patients and CDI patients.

Bacterial diversity was decreased in CDC and CDI patients, and microbiota composition was mostly patient group-specific. Interestingly, microbiota composition was associated with previous antibiotic use within the control group, but not within the CDC group. This may suggest that CDC patients already have a disturbed bacterial community prior to colonization, independent of antibiotic treatment, although the underlying reason remains unclear. Another explanation could be that, as 73.2% of CDC patients had previous antibiotic use, too few CDC patients without antibiotic use were included to effectively identify an antibiotic treatment effect within this group.

Multiple differentially abundant genera were found between control, CDC patients, and CDI patients, and included *Eubacterium hallii*, *Fusicatenibacter*, and *Veillonella*. Bacterial network analysis showed that *C. difficile* was directly negatively associated with *Fusicatenibacter* in CDI patients, and directly positively associated with *Veillonella* in CDC patients. This may indicate that *Fusicatenibacter* may play a role in preventing CDI development, while *Veillonella* may play a role in *C. difficile* colonization, respectively.

It has previously been hypothesized that *Eubacterium* species are protective against CDI development in asymptomatic carriers [10]. In our study, *E. hallii* was more abundant in controls (with and without antibiotic use) than in CDC patients. *E. hallii* is known to produce the three main SCFAs (propionate, acetate, and butyrate [29,30]), and is increasingly being investigated for its potential benefit in metabolic disease [31]. This bacterium may contribute to colonization resistance against *C. difficile* through SCFAs production, although the role of SCFAs against *C. difficile* remains debated [32,33]. Possibly, *E. hallii* contributes to colonization resistance through secondary bile-acid production. Secondary bile acids are known to inhibit *C. difficile* growth, and a secondary bile acid-producing bacterium, *Clostridium scindens*, enhances colonization resistance against *C. difficile* [34,35]. *E. hallii* possesses *bsh* genes, which are necessary for deconjugation of conjugated bile acids, which is a crucial step prior to converting deconjugated bile acids into secondary bile acids [31]. However, although the most important enzyme for secondary bile acids conversion, 7α-dehydroxylase, was demonstrated to be present in *Eubacterium* species, no homologue has been detected in *E. hallii*’s genome [31,36,37,38].

*Veillonella* was more abundant in CDI patients than in CDC patients and controls with prior antibiotic use, and was positively associated with *Clostridioides* in colonized patients in our study. *Veillonella* is normally found in the oral cavity, where it can form dental plaques with *Streptococcus*, but is also found in atherosclerotic plaques and fecal samples from patients with atherosclerosis [39,40]. *Veillonella and* streptococci may be metabolically linked through lactic acid, which also holds for other lactic-acid producing bacteria, like lactobacilli [41,42]. *Lactobacillus* and *Veillonella* were indeed directly positively linked in our network analysis. While increased relative abundance of *Veillonella* may be a result of intrinsic resistance to multiple antibiotics, a recent in vitro study showed that *Veillonella* increases when a dysbiotic microbiota is co-cultivated with *C. difficile* [43]. In addition, increased *Veillonella* abundance has been reported prior to CDI onset [44]. These studies, combined with our data, suggest that *Veillonella* is associated with *C. difficile* colonization and infection. It remains unclear whether *Veillonella* has a role in CDI development (e.g., via biofilm formation), or whether it simply outgrows as a result of altered metabolic pathways or unoccupied niches in the gut due to antibiotic use or *C. difficile* expansion.

*Fusicatenibacter* was differentially abundant between C-AB and CDC patients, and was negatively associated with *Clostridioides* in CDI patients in our study. This bacterium has only been cultured recently (2013), and we are the first to describe an association between *Fusicatenibacter* and *C. difficile* colonization or infection [45]. Previously, *Fusicatenibacter sacchivorans*, the only known species within the *Fusicatenibacter* genus, was shown to be increased in inactive ulcerative colitis (UC) patients and decreased in active UC, related to its positive association with IL-10 production [46].

Our study had some limitations. Almost all diagnosed CDI patients (39 of 41) came from the LUMC, while CDC and controls were derived from both Amphia hospital and LUMC. As the LUMC is a university affiliated hospital instead of a general hospital, patient characteristics in these groups may not have been completely comparable. As such, solid organ transplants, previous hospitalization, immunosuppressant use, and chemotherapy were more frequent in LUMC. Several of these clinical variables significantly affected overall microbiota composition, which challenges studying the sole effect of CDI on microbiota composition. Another limitation is that a single stool sample was available. Therefore, it is impossible to determine if patients were transiently or persistently colonized by *C. difficile*. Patients classified as CDC might have included patients with only transient passage of spores [2]. Lastly, we have not performed functional characterization of the microbiota, e.g., by metabolomics or transcriptomics.

However, our study had multiple important strengths. Firstly, this is the first study that investigates microbiota composition of *C. difficile* colonized patients, as compared to controls and CDI patients, with more than 10 patients included per group. This allowed for more robust statistical analysis, and for detecting smaller and subtle changes within the composition. Secondly, controls in this study were not healthy controls. Instead, controls and CDC patients were selected from the same cohort of newly admitted patients, and all three groups were hospitalized on the same wards to make the comparisons more fair. Thirdly, CDI was well defined. Although molecular testing is nowadays often used as a stand-alone test to diagnose CDI, these assays cannot discern colonization from infection [47]. In our study, all samples suspected of CDI were (also) tested with an assay detecting free toxins. Laboratory results were interpreted in conjunction with clinical symptoms. According to the Dutch sentinel surveillance program and the ECDC criteria, patients had to have diarrhea for at least 2 days and/or pseudomembranous colitis at endoscopy and no other apparent cause of diarrhea. Although milder cases may have been missed by using these strict criteria, we are quite confident that our CDI group consisted of clinical relevant CDI cases requiring CDI treatment. Fourthly, duplicates for DNA extraction and sequencing were included to detect potential bias. All these duplicates showed very high similarity in composition profiles, demonstrating the reproducibility of DNA extraction and sequencing procedures (Figure S1).

## 5. Conclusions

We demonstrated that colonization and infection by *C. difficile* are associated with decreased bacterial diversity in the gut and differences in relative abundance of specific bacterial taxa, including *Veillonella*, *Fusicatenibacter*, *Eubacterium hallii*, *Bacteroides*, and members of the *Lachnospiraceae* and *Ruminococcaceae* families. Future studies could focus on functional characterization of the microbiota, e.g., by metabolomics or transcriptomics, and on co-cultivation of specific bacteria, e.g., *Fusicatenibacter* with *C. difficile*, in light of *C. difficile* colonization and infection. In addition, it is relevant to determine if the observed gut microbiota changes are present before acquiring colonization and/or CDI, or merely as a consequence.

## Figures and Tables

**Table 1:** Subject characteristics.

	CDI Patients (*n* = 41)	CDC Patients (*n* = 41)	Control Patients (*n* = 43)	*p*-Value
**Age in years, mean (SD)**	57.5 (17.6)	55.3 (18.7)	57.8 (13.5)	0.76
**Sex**				0.47
Male	22/41 (53.7%)	22/41 (53.7%)	28/43 (65.1%)	
Female	19/41 (46.3%)	19/41 (46.3%)	15/43 (34.9%)	
**Previous CDI**				
Last 8 weeks	7/41 (17.1%)	1/41 (2.4%)	0/42 (0%)	0.003
>8 weeks earlier	7/41 (17.1%)	3/41 (7.3%)	0/42 (0%)	0.02
**Current CDI episode**				
Primary episode	30/41 (73.2%)			
Persistent primary episode	2/41 (4.9%)			
1st recurrence of primary episode	3/41 (7.3%)			
2nd recurrence of primary episode	1/41 (2.4%)			
5th recurrence of primary episode	1/41 (2.4%)			
1st reinfection	1/41 (2.4%)			
2nd reinfection	2/41 (4.9%)			
2nd recurrence of first reinfection	1/41 (2.4%)			
**Previous hospitalization (last year)**	29/41 (70.7%)	30/41 (73.2%)	19/42 (45.2%)	0.01
**Comorbidities**				
IBD	2/41 (4.9%)	7/41 (17.1%)	2/42 (4.8%)	0.08
Solid organ transplant	17/41 (41.5%)	9/41 (22.0%)	2/42 (4.8%)	<0.001
Solid malignancy	5/41 (12.2%)	6/41 (14.6%)	11/42 (26.2%)	0.2
Hematological malignancy	9/41 (22.0%)	0/41 (0%)	2/42 (4.8%)	0.001
**Previous medication use (last 3 months)**				
Antibiotics	40/41 (97.6%)	30/41 (73.2%)	25/42 (59.5%)	<0.001
Immunosuppressants	30/41 (73.2%)	17/41 (41.5%)	13/42 (31.0%)	<0.001
Chemotherapy	10/41 (24.4%)	2/41 (4.9%)	5/42 (11.9%)	0.03
PPI or antacids	31/41 (75.6%)	30/41 (73.2%)	19/42 (45.2%)	0.006

## Supplementary Materials

The following are available online at https://www.mdpi.com/2076-2607/8/5/677/s1.

## References

[1] Smits W.K., Lyras D., Lacy D.B., Wilcox M.H., Kuijper E.J. (2016). *Clostridium difficile* infection. Nat. Rev. Dis. Primers.

[2] Crobach M.J.T., Vernon J.J., Loo V.G., Kong L.Y., Pechine S., Wilcox M.H., Kuijper E.J. (2018). Understanding *Clostridium difficile* colonization. Clin. Microbiol. Rev..

[3] Eyre D.W., Griffiths D., Vaughan A., Golubchik T., Acharya M., O’Conor L., Crook D.W., Walker A.S., Peto T.E.A. (2013). Asymptomatic *Clostridium difficile* colonisation and onward transmission. PLoS ONE.

[4] Kong L.Y., Eyre D.W., Corbeil J., Raymond F., Walker A.S., Wilcox M.H., Crook D.W., Michaud S., Toye B., Frost E. (2019). *Clostridium difficile*: Investigating transmission patterns between infected and colonized patients using whole genome sequencing. Clin. Infect. Dis..

[5] Zacharioudakis I.M., Zervou F.N., Pliakos E.E., Ziakas P.D., Mylonakis E. (2015). Colonization with toxinogenic *C. difficile* upon hospital admission, and risk of infection: A systematic review and meta-analysis. Am. J. Gastroenterol..

[6] Blixt T., Gradel K.O., Homann C., Seidelin J.B., Schonning K., Lester A., Houlind J., Stangerup M., Gottlieb M., Knudsen J.D. (2017). Asymptomatic carriers contribute to nosocomial *Clostridium difficile* infection: A cohort study of 4508 patients. Gastroenterology.

[7] Rodriguez C., Romero E., Garrido-Sanchez L., Alcain-Martinez G., Andrade R.J., Taminiau B., Daube G., Garcia-Fuentes E. (2020). Microbiota insights in *Clostridium difficile* infection and inflammatory bowel disease. Gut Microbes.

[8] Zhang L., Dong D., Jiang C., Li Z., Wang X., Peng Y. (2015). Insight into alteration of gut microbiota in *Clostridium difficile* infection and asymptomatic *C. difficile* colonization. Anaerobe.

[9] Antharam V.C., Li E.C., Ishmael A., Sharma A., Mai V., Rand K.H., Wang G.P. (2013). Intestinal dysbiosis and depletion of butyrogenic bacteria in *Clostridium difficile* infection and nosocomial diarrhea. J. Clin. Microbiol..

[10] Vincent C., Miller M.A., Edens T.J., Mehrotra S., Dewar K., Manges A.R. (2016). Bloom and bust: Intestinal microbiota dynamics in response to hospital exposures and *Clostridium difficile* colonization or infection. Microbiome.

[11] Robinson J.I., Weir W.H., Crowley J.R., Hink T., Reske K.A., Kwon J.H., Burnham C.D., Dubberke E.R., Mucha P.J., Henderson J.P. (2019). Metabolomic networks connect host-microbiome processes to human *Clostridioides difficile* infections. J. Clin. Invest..

[12] Vendrik K.E.W., Crobach M.J.T., Terveer E.M., Harmanus C., Sanders I.M.J.G., Kuijper E.J., Notermans D.W., De Greeff S.C., Alblas J., Van Dissel J.T. (2018). Twelfth Annual Report of the National Reference Laboratory for Clostridium difficile and Results of the Sentinel Surveillance May 2017—May 2018.

[13] European Centre for Disease Prevention and Control (2017). European Surveillance of Clostridium difficile Infections.

[14] Paltansing S., van den Berg R.J., Guseinova R.A., Visser C.E., van der Vorm E.R., Kuijper E.J. (2007). Characteristics and incidence of *Clostridium difficile*-associated disease in The Netherlands, 2005. Clin. Microbiol. Infect..

[15] Persson S., Torpdahl M., Olsen K.E. (2008). New multiplex PCR method for the detection of *Clostridium difficile* toxin A (tcdA) and toxin B (tcdB) and the binary toxin (cdtA/cdtB) genes applied to a Danish strain collection. Clin. Microbiol. Infect..

[16] Terveer E.M., Crobach M.J., Sanders I.M., Vos M.C., Verduin C.M., Kuijper E.J. (2017). Detection of *Clostridium difficile* in feces of asymptomatic patients admitted to the hospital. J. Clin. Microbiol..

[17] Fawley W.N., Knetsch C.W., MacCannell D.R., Harmanus C., Du T., Mulvey M.R., Paulick A., Anderson L., Kuijper E.J., Wilcox M.H. (2015). Development and validation of an internationally-standardized, high-resolution capillary gel-based electrophoresis PCR-ribotyping protocol for *Clostridium difficile*. PLoS ONE.

[18] Ramiro-Garcia J., Hermes G.D.A., Giatsis C., Sipkema D., Zoetendal E.G., Schaap P.J., Smidt H. (2016). NG-Tax, a highly accurate and validated pipeline for analysis of 16S rRNA amplicons from complex biomes [version 1; referees: 2 approved with reservations, 1 not approved]. F1000 Res..

[19] Quast C., Pruesse E., Yilmaz P., Gerken J., Schweer T., Yarza P., Peplies J., Glockner F.O. (2013). The SILVA ribosomal RNA gene database project: Improved data processing and web-based tools. Nucleic Acids Res..

[20] Ducarmon Q.R., Hornung B.V.H., Geelen A.R., Kuijper E.J., Zwittink R.D. (2020). Toward standards in clinical microbiome studies: Comparison of three DNA extraction methods and two bioinformatic pipelines. mSystems.

[21] Bokulich N.A., Subramanian S., Faith J.J., Gevers D., Gordon J.I., Knight R., Mills D.A., Caporaso J.G. (2013). Quality-filtering vastly improves diversity estimates from Illumina amplicon sequencing. Nat. Methods.

[22] Kurtz Z.D., Muller C.L., Miraldi E.R., Littman D.R., Blaser M.J., Bonneau R.A. (2015). Sparse and compositionally robust inference of microbial ecological networks. PLoS Comput. Biol..

[23] Lahti L., Shetty S. (2017). Tools for Microbiome Analysis in R; Microbiome package version 1.2.1. https://microbiome.github.io/.

[24] Love M.I., Huber W., Anders S. (2014). Moderated estimation of fold change and dispersion for RNA-seq data with DESeq2. Genome Biol..

[25] McMurdie P.J., Holmes S. (2013). phyloseq: An R package for reproducible interactive analysis and graphics of microbiome census data. PLoS ONE.

[26] Oksanen J.F., Blanchet G.F., Friendly M., Kindt R., Legendre P., McGlinn D., Minchin P.R., O’Hara R.B., Simpson G.L., Solymos P. Vegan: Community Ecology Package. R package version 2.5-2. https://CRAN.R-project.org/package=vegan.

[27] Wickham H. (2009). ggplot2: Elegant Graphics for Data Analysis.

[28] Shannon P., Markiel A., Ozier O., Baliga N.S., Wang J.T., Ramage D., Amin N., Schwikowski B., Ideker T. (2003). Cytoscape: A software environment for integrated models of biomolecular interaction networks. Genome Res..

[29] Engels C., Ruscheweyh H.J., Beerenwinkel N., Lacroix C., Schwab C. (2016). The common gut microbe *Eubacterium hallii* also contributes to intestinal propionate formation. Front. Microbiol..

[30] Duncan S.H., Louis P., Flint H.J. (2004). Lactate-utilizing bacteria, isolated from human feces, that produce butyrate as a major fermentation product. Appl. Environ. Microbiol..

[31] Udayappan S., Manneras-Holm L., Chaplin-Scott A., Belzer C., Herrema H., Dallinga-Thie G.M., Duncan S.H., Stroes E.S.G., Groen A.K., Flint H.J. (2016). Oral treatment with *Eubacterium hallii* improves insulin sensitivity in db/db mice. NPJ Biofilms Microbiomes..

[32] Theriot C.M., Koenigsknecht M.J., Carlson P.E., Hatton G.E., Nelson A.M., Li B., Huffnagle G.B., J Z.L., Young V.B. (2014). Antibiotic-induced shifts in the mouse gut microbiome and metabolome increase susceptibility to *Clostridium difficile* infection. Nat Commun..

[33] Lawley T.D., Clare S., Walker A.W., Stares M.D., Connor T.R., Raisen C., Goulding D., Rad R., Schreiber F., Brandt C. (2012). Targeted restoration of the intestinal microbiota with a simple, defined bacteriotherapy resolves relapsing *Clostridium difficile* disease in mice. PLoS Pathog..

[34] Buffie C.G., Bucci V., Stein R.R., McKenney P.T., Ling L., Gobourne A., No D., Liu H., Kinnebrew M., Viale A. (2015). Precision microbiome reconstitution restores bile acid mediated resistance to *Clostridium difficile*. Nature.

[35] Sorg J.A., Sonenshein A.L. (2010). Inhibiting the initiation of *Clostridium difficile* spore germination using analogs of chenodeoxycholic acid, a bile acid. J. Bacteriol..

[36] Doerner K.C., Takamine F., LaVoie C.P., Mallonee D.H., Hylemon P.B. (1997). Assessment of fecal bacteria with bile acid 7 alpha-dehydroxylating activity for the presence of bai-like genes. Appl. Environ. Microbiol..

[37] Shetty S.A., Ritari J., Paulin L., Smidt H., De Vos W.M. (2017). Complete Genome Sequence of *Eubacterium hallii* Strain L2–7. Genome Announc..

[38] Takamine F., Imamura T. (1995). Isolation and characterization of bile acid 7-dehydroxylating bacteria from human feces. Microbiol. Immunol..

[39] Mikx F.H., van der Hoeven J.S., Konig K.G., Plasschaert A.J., Guggenheim B. (1972). Establishment of defined microbial ecosystems in germ-free rats. I. The effect of the interactions of streptococcus mutans or *Streptococcus sanguis* with *Veillonella alcalescens* on plaque formation and caries activity. Caries Res..

[40] Koren O., Spor A., Felin J., Fak F., Stombaugh J., Tremaroli V., Behre C.J., Knight R., Fagerberg B., Ley R.E. (2011). Human oral, gut, and plaque microbiota in patients with atherosclerosis. Proc. Natl. Acad. Sci. USA.

[41] Mashima I., Nakazawa F. (2014). The influence of oral *Veillonella* species on biofilms formed by *Streptococcus* species. Anaerobe.

[42] Van den Bogert B., Erkus O., Boekhorst J., de Goffau M., Smid E.J., Zoetendal E.G., Kleerebezem M. (2013). Diversity of human small intestinal *Streptococcus* and *Veillonella* populations. FEMS Microbiol. Ecol..

[43] Horvat S., Rupnik M. (2018). Interactions Between *Clostridioides difficile* and fecal Microbiota in In Vitro batch model: Growth, sporulation, and microbiota changes. Front. Microbiol..

[44] Khanna S., Montassier E., Schmidt B., Patel R., Knights D., Pardi D.S., Kashyap P. (2016). Gut microbiome predictors of treatment response and recurrence in primary *Clostridium difficile* infection. Aliment Pharmacol. Ther..

[45] Takada T., Kurakawa T., Tsuji H., Nomoto K. (2013). *Fusicatenibacter saccharivorans* gen. nov., sp. nov., isolated from human faeces. Int. J. Syst. Evol. Microbiol..

[46] Takeshita K., Mizuno S., Mikami Y., Sujino T., Saigusa K., Matsuoka K., Naganuma M., Sato T., Takada T., Tsuji H. (2016). A single species of *Clostridium* subcluster XIVa decreased in ulcerative colitis patients. Inflamm. Bowel Dis..

[47] Crobach M.J., Planche T., Eckert C., Barbut F., Terveer E.M., Dekkers O.M., Wilcox M.H., Kuijper E.J. (2016). European Society of Clinical Microbiology and Infectious Diseases: Update of the diagnostic guidance document for *Clostridium difficile* infection. Clin. Microbiol. Infect..

