# Supplementary material for: The Bacterial Gut Microbiota of Adult Patients Infected, Colonized or Noncolonized by Clostridioides difficile
Source: Microorganisms. 2020 May 6;8(5):677. doi: 10.3390/microorganisms8050677 (PMC7284656; doi:10.3390/microorganisms8050677)
Supplement: Supplementary file 1 [file microorganisms-08-00677-s001.zip › microorganisms-795617-supplementary-proofreading/Figure_S1_Technical_Replicates.pdf]

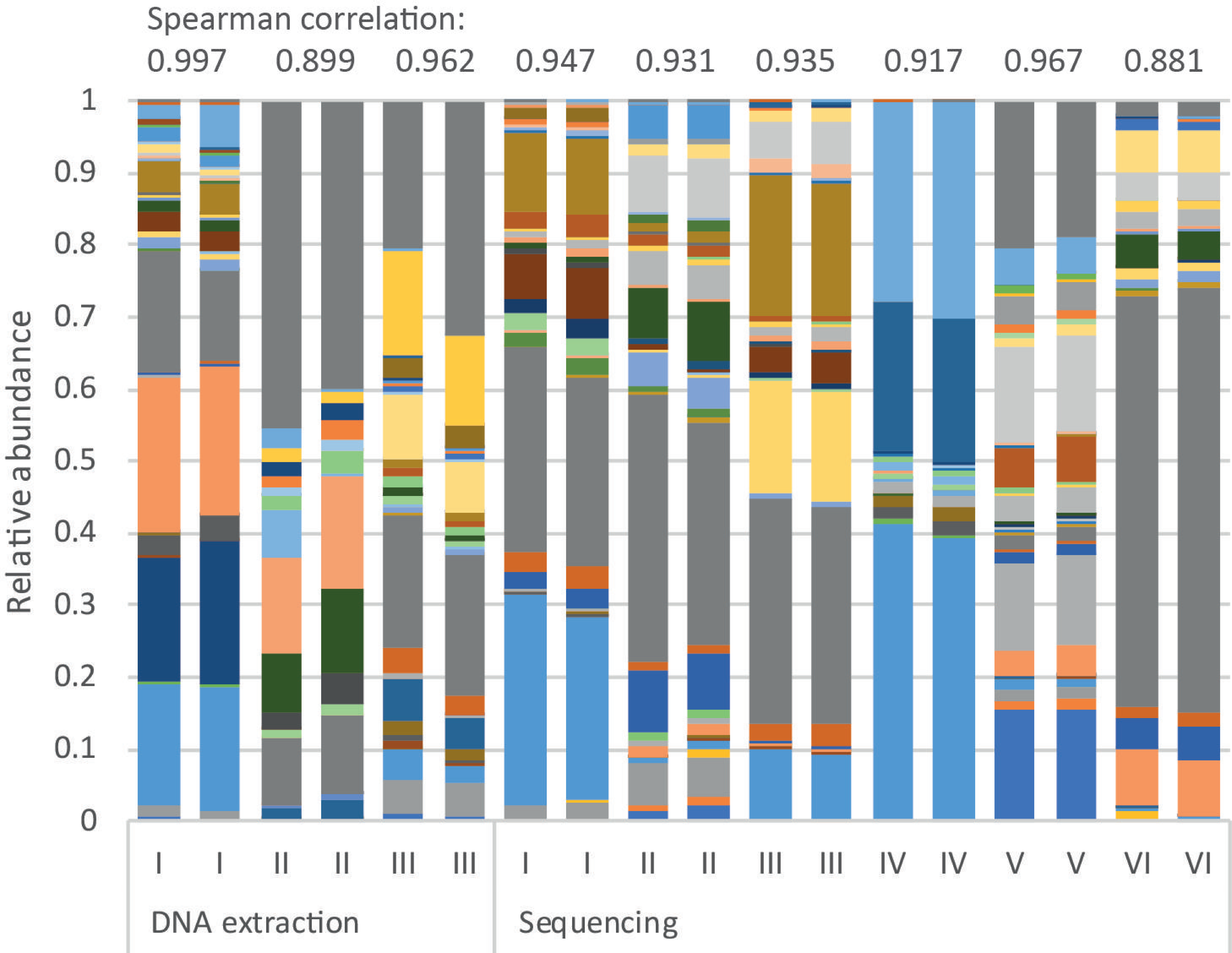
Spearman correlation:
0.997
0.899
0.962
0.947
0.931
0.935
0.917
0.967
0.881
Relative abundance
1
0.9
0.8
0.7
0.6
0.5
0.4
0.3
0.2
0.1
0
I
I
II
II
III
III
I
I
II
II
III
III
IV
IV
V
V
VI
VI
DNA extraction
Sequencing
