# Supplementary material for: The Bacterial Gut Microbiota of Adult Patients Infected, Colonized or Noncolonized by Clostridioides difficile
Source: Microorganisms. 2020 May 6;8(5):677. doi: 10.3390/microorganisms8050677 (PMC7284656; doi:10.3390/microorganisms8050677)
Supplement: Supplementary file 1 [file microorganisms-08-00677-s001.zip › microorganisms-795617-supplementary-proofreading/Table_S1_PERMANOVA.pdf]

Separate PERMANOVA testing for clinical variables

Clinical variable	P-value	R^2	How many samples "uncertain"
Category	0.001	0.075	not applicable
2nd_cephalosporins	0.001	0.0468	1
pAB	0.001	0.03961	13
metronidazole	0.001	0.0321	1
carbapenems	0.001	0.02955	1
vancomycin	0.003	0.02633	1
3rd_cephalosporins	0.02	0.02351	1
clindamycin	0.025	0.02241	1
Immuno_suppressants	0.001	0.02082	not applicable
solid_organ_transplant	0.005	0.01615	not applicable
previous_CDI_within_previous_8_weeks	0.008	0.01584	not applicable
PPI_or_antacids	0.017	0.01426	not applicable
previous_CDI_morethan_8.weeks	0.025	0.01362	not applicable
