# Supplementary material for: The Bacterial Gut Microbiota of Adult Patients Infected, Colonized or Noncolonized by Clostridioides difficile
Source: Microorganisms. 2020 May 6;8(5):677. doi: 10.3390/microorganisms8050677 (PMC7284656; doi:10.3390/microorganisms8050677)
Supplement: Supplementary file 1 [file microorganisms-08-00677-s001.zip › microorganisms-795617-supplementary-proofreading/Table_S2A_Differential_Abundance.pdf]

	OTU number	Phylum	Genus	log_2Fold Change	Adjusted p-value
	1648581007	p__Firmicutes	g__[Eubacterium]_hallii_group	3.84	4.73E-02
	1648581942	p__Firmicutes	g__Fusicatenibacter	2.97	1.72E-02
	1648581841	p__Firmicutes	g__Flavonifractor	-2.88	1.91E-02
	1648588	p__Firmicutes	g__Enterococcus	-4.18	5.94E-08
	164858332	p__Firmicutes	g__[Clostridium]_innocuum_group	-4.31	2.29E-05
	164858777	p__Firmicutes	g__Enterococcus	-4.42	3.87E-02
	1648581119	p__Firmicutes	g__Lachnoclostridium	-4.43	1.70E-02
	164858931	p__Firmicutes	g__[Ruminococcus]_gnavus_group	-4.44	3.02E-06
	16485812	p__Firmicutes	g__Lachnoclostridium	-4.63	2.22E-02
	164858201	p__Firmicutes	g__Enterococcus	-4.64	2.87E-02
C-AB versus CDC	164858382	p__Firmicutes	g__Lachnoclostridium	-4.68	4.73E-02
	164858286	p__Firmicutes	g__Enterococcus	-4.69	1.91E-02
	1648582	p__Firmicutes	g__Sellimonas	-4.82	2.35E-03
	1648585657	p__Firmicutes	g__Enterococcus	-4.84	2.87E-02
	164858293	p__Firmicutes	g__Enterococcus	-4.98	3.86E-02
	1648581669	p__Firmicutes	g__Enterococcus	-5.03	4.73E-02
	1648581801	p__Firmicutes	g__[Ruminococcus]_gnavus_group	-5.08	8.90E-04
	1648580	p__Firmicutes	g__Lachnoclostridium	-5.11	1.55E-07
	164858516	p__Firmicutes	g__Lactobacillus	-5.24	2.90E-02
	1648582959	p__Firmicutes	g__Enterococcus	-5.60	1.72E-02
	164858257	p__Firmicutes	g__Enterococcus	-5.66	1.70E-02
	1648581238	p__Firmicutes	g__Clostridioides	-5.68	9.00E-06
	164858150	p__Firmicutes	g__Enterococcus	-6.48	8.90E-04
	16485877	p__Firmicutes	g__Blautia	-7.74	3.02E-06
	1648582328	p__Firmicutes	g__uncultured	-9.48	1.55E-07
C+AB vs CDC	1648581007	p__Firmicutes	g__[Eubacterium]_hallii_group	6.61	1.38E-02
	1648581238	p__Firmicutes	g__Clostridioides	-3.91	1.38E-02
	1648581792	p__Actinobacteria	g__Collinsella	8.53	1.38E-08
	1648582527	p__Actinobacteria	g__Bifidobacterium	8.18	3.38E-08
	1648582144	p__Firmicutes	g__Ruminococcus_2	7.33	8.76E-07
	1648581151	p__Firmicutes	g__Roseburia	5.85	1.07E-03
	1648582610	p__Firmicutes	g__Ruminococcus_2	5.47	6.32E-03
	16485811575	p__Firmicutes	g__Clostridium_sensu_stricto_1	5.32	3.26E-02
	1648588051	p__Firmicutes	g__[Ruminococcus]_torques_group	4.88	2.49E-02
	16485817	p__Firmicutes	g__Blautia	4.27	1.77E-02
	1648582252	p__Firmicutes	g__Romboutsia	4.18	1.77E-02
C+AB vs CDI	164858191	p__Firmicutes	g__Blautia	2.73	3.95E-02
	164858296	p__Firmicutes	g__Blautia	2.70	3.47E-02
	16485813	p__Firmicutes	g__Blautia	2.41	2.97E-03
	164858931	p__Firmicutes	g__[Ruminococcus]_gnavus_group	-2.66	8.24E-03
	1648581841	p__Firmicutes	g__Flavonifractor	-2.86	3.86E-02
	16485814	p__Firmicutes	g__Lachnoclostridium	-2.99	1.81E-02
	1648581669	p__Firmicutes	g__Enterococcus	-3.65	1.92E-02
	164858467	p__Firmicutes	g__Veillonella	-3.75	1.77E-02
	1648582163	p__Bacteroidetes	g__Bacteroides	-3.94	6.32E-03
	1648585657	p__Firmicutes	g__Enterococcus	-5.05	1.77E-02

	1648581238	p__Firmicutes	g__Clostridioides	-6.45	1.27E-14
	164858244	p__Firmicutes	g__Erysipelotrichaceae_UCG-003	11.10	7.21E-16
	1648581792	p__Actinobacteria	g__Collinsella	9.58	6.64E-20
	1648582610	p__Firmicutes	g__Ruminococcus_2	8.65	6.05E-08
	1648582527	p__Actinobacteria	g__Bifidobacterium	7.91	7.47E-16
	1648582144	p__Firmicutes	g__Ruminococcus_2	6.90	4.10E-10
	16485811575	p__Firmicutes	g__Clostridium_sensu_stricto_1	6.33	1.97E-06
	1648581879	p__Euryarchaeota	g__Methanobrevibacter	5.62	1.90E-05
	1648581785	p__Firmicutes	g__uncultured	4.77	1.95E-05
	1648581991	p__Firmicutes	g__Subdoligranulum	4.62	1.10E-06
	1648581796	p__Firmicutes	g__Coprococcus_1	4.29	1.27E-04
	1648582252	p__Firmicutes	g__Romboutsia	4.28	9.23E-06
	1648582992	p__Firmicutes	g__Blautia	4.01	1.11E-03
	16485810430	p__Firmicutes	g__Roseburia	3.83	2.76E-03
	16485817	p__Firmicutes	g__Blautia	3.77	4.88E-05
	164858191	p__Firmicutes	g__Blautia	3.65	1.56E-06
	1648588054	p__Firmicutes	g__Lachnospiraceae_ND3007_group	3.52	1.50E-02
	1648585	p__Firmicutes	g__[Ruminococcus]_gauvreauii_group	3.24	3.46E-02
CDC vs CDI	1648581355	p__Firmicutes	g__Anaerostipes	2.45	2.23E-02
	1648582724	p__Actinobacteria	g__Bifidobacterium	2.41	8.31E-03
	164858296	p__Firmicutes	g__Blautia	2.35	2.81E-03
	164858888	p__Firmicutes	g__Blautia	2.28	1.96E-02
	16485813	p__Firmicutes	g__Blautia	2.26	1.92E-05
	1648581980	p__Firmicutes	g__Blautia	2.21	2.34E-02
	164858216	p__Firmicutes	g__Blautia	2.15	2.23E-02
	164858927	p__Firmicutes	g__Blautia	2.11	2.29E-02
	1648581996	p__Firmicutes	g__Blautia	2.06	4.37E-02
	1648581866	p__Firmicutes	g__Blautia	2.02	3.01E-02
	1648581069	p__Firmicutes	g__Blautia	2.02	3.41E-02
	164858377	p__Firmicutes	g__Blautia	2.00	3.57E-02
	1648581900	p__Firmicutes	g__Blautia	1.99	4.85E-02
	16485879	p__Firmicutes	g__Streptococcus	1.97	1.87E-03
	1648581880	p__Firmicutes	g__Blautia	1.94	4.55E-02
	164858499	p__Firmicutes	g__Erysipelatoclostridium	-2.11	2.15E-02
	1648582163	p__Bacteroidetes	g__Bacteroides	-2.35	3.46E-02
	1648581238	p__Firmicutes	g__Clostridioides	-2.49	6.43E-03
	1648584047	p__Firmicutes	g__Lactobacillus	-2.84	4.25E-02
	1648581787	p__Bacteroidetes	g__Bacteroides	-3.10	3.01E-02
	1648584	p__Bacteroidetes	g__Bacteroides	-3.33	1.17E-04
	164858467	p__Firmicutes	g__Veillonella	-3.41	1.00E-03
