# Supplementary material for: The Bacterial Gut Microbiota of Adult Patients Infected, Colonized or Noncolonized by Clostridioides difficile
Source: Microorganisms. 2020 May 6;8(5):677. doi: 10.3390/microorganisms8050677 (PMC7284656; doi:10.3390/microorganisms8050677)
Supplement: Supplementary file 1 [file microorganisms-08-00677-s001.zip › microorganisms-795617-supplementary-proofreading/Table_S2B_Relative_Abundance.pdf]

OTU	Phylum	Genus	Category_extra	Abundance in %	Abundance	sd
1648580	p__Firmicutes	g__Lachnoclostridium	C-AB	0.04	0.0004	0.001
1648580	p__Firmicutes	g__Lachnoclostridium	C+AB	2.36	0.0236	0.0825
1648580	p__Firmicutes	g__Lachnoclostridium	CDC	1.7	0.017	0.0519
1648580	p__Firmicutes	g__Lachnoclostridium	CDI	1.94	0.0194	0.0279
1648581007	p__Firmicutes	g__[Eubacterium]_hallii_group	C-AB	0.6	0.006	0.0058
1648581007	p__Firmicutes	g__[Eubacterium]_hallii_group	C+AB	0.59	0.0059	0.0099
1648581007	p__Firmicutes	g__[Eubacterium]_hallii_group	CDC	0.05	0.0005	0.0025
1648581007	p__Firmicutes	g__[Eubacterium]_hallii_group	CDI	0.04	0.0004	0.0015
16485810430	p__Firmicutes	g__Roseburia	C-AB	0.29	0.0029	0.0055
16485810430	p__Firmicutes	g__Roseburia	C+AB	0.06	0.0006	0.0013
16485810430	p__Firmicutes	g__Roseburia	CDC	0.24	0.0024	0.0076
16485810430	p__Firmicutes	g__Roseburia	CDI	0.17	0.0017	0.0102
1648581069	p__Firmicutes	g__Blautia	C-AB	0.16	0.0016	0.0013
1648581069	p__Firmicutes	g__Blautia	C+AB	0.12	0.0012	0.0013
1648581069	p__Firmicutes	g__Blautia	CDC	0.15	0.0015	0.0019
1648581069	p__Firmicutes	g__Blautia	CDI	0.04	0.0004	0.001
1648581119	p__Firmicutes	g__Lachnoclostridium	C-AB	0.02	0.0002	0.0005
1648581119	p__Firmicutes	g__Lachnoclostridium	C+AB	0.17	0.0017	0.003
1648581119	p__Firmicutes	g__Lachnoclostridium	CDC	0.39	0.0039	0.0101
1648581119	p__Firmicutes	g__Lachnoclostridium	CDI	0.26	0.0026	0.0085
1648581151	p__Firmicutes	g__Roseburia	C-AB	0.58	0.0058	0.0097
1648581151	p__Firmicutes	g__Roseburia	C+AB	0.68	0.0068	0.013
1648581151	p__Firmicutes	g__Roseburia	CDC	0.17	0.0017	0.0032
1648581151	p__Firmicutes	g__Roseburia	CDI	0.02	0.0002	0.0008
16485811575	p__Firmicutes	g__Clostridium_sensu_stricto_1	C-AB	0.36	0.0036	0.0098
16485811575	p__Firmicutes	g__Clostridium_sensu_stricto_1	C+AB	0.57	0.0057	0.0184
16485811575	p__Firmicutes	g__Clostridium_sensu_stricto_1	CDC	0.26	0.0026	0.0076
16485811575	p__Firmicutes	g__Clostridium_sensu_stricto_1	CDI	0.03	0.0003	0.0015
16485812	p__Firmicutes	g__Lachnoclostridium	C-AB	0	0	0.0001
16485812	p__Firmicutes	g__Lachnoclostridium	C+AB	0.16	0.0016	0.005
16485812	p__Firmicutes	g__Lachnoclostridium	CDC	0.12	0.0012	0.0034
16485812	p__Firmicutes	g__Lachnoclostridium	CDI	0.18	0.0018	0.0024
1648581238	p__Firmicutes	g__Clostridioides	C-AB	0	0	0.0001
1648581238	p__Firmicutes	g__Clostridioides	C+AB	0.05	0.0005	0.0019
1648581238	p__Firmicutes	g__Clostridioides	CDC	0.7	0.007	0.0221
1648581238	p__Firmicutes	g__Clostridioides	CDI	2.49	0.0249	0.0295
16485813	p__Firmicutes	g__Blautia	C-AB	9.77	0.0977	0.0837
16485813	p__Firmicutes	g__Blautia	C+AB	10.71	0.1071	0.0881
16485813	p__Firmicutes	g__Blautia	CDC	11.06	0.1106	0.1358
16485813	p__Firmicutes	g__Blautia	CDI	2.54	0.0254	0.0543
1648581355	p__Firmicutes	g__Anaerostipes	C-AB	1.84	0.0184	0.0183
1648581355	p__Firmicutes	g__Anaerostipes	C+AB	0.71	0.0071	0.016
1648581355	p__Firmicutes	g__Anaerostipes	CDC	0.54	0.0054	0.0125
1648581355	p__Firmicutes	g__Anaerostipes	CDI	0.11	0.0011	0.0037
16485814	p__Firmicutes	g__Lachnoclostridium	C-AB	0.02	0.0002	0.0006
16485814	p__Firmicutes	g__Lachnoclostridium	C+AB	0.15	0.0015	0.0039

16485814	p__Firmicutes	g__Lachnoclostridium	CDC	0.09	0.0009	0.0017
16485814	p__Firmicutes	g__Lachnoclostridium	CDI	0.47	0.0047	0.0074
164858150	p__Firmicutes	g__Enterococcus	C-AB	0	0	0.0001
164858150	p__Firmicutes	g__Enterococcus	C+AB	0.1	0.001	0.0021
164858150	p__Firmicutes	g__Enterococcus	CDC	0.14	0.0014	0.0031
164858150	p__Firmicutes	g__Enterococcus	CDI	0.21	0.0021	0.0032
1648581669	p__Firmicutes	g__Enterococcus	C-AB	0	0	0
1648581669	p__Firmicutes	g__Enterococcus	C+AB	0.04	0.0004	0.0009
1648581669	p__Firmicutes	g__Enterococcus	CDC	0.05	0.0005	0.0011
1648581669	p__Firmicutes	g__Enterococcus	CDI	0.71	0.0071	0.0192
16485817	p__Firmicutes	g__Blautia	C-AB	2.89	0.0289	0.033
16485817	p__Firmicutes	g__Blautia	C+AB	2.14	0.0214	0.0478
16485817	p__Firmicutes	g__Blautia	CDC	1.21	0.0121	0.0283
16485817	p__Firmicutes	g__Blautia	CDI	0.09	0.0009	0.0035
1648581785	p__Firmicutes	g__uncultured	C-AB	0.49	0.0049	0.0065
1648581785	p__Firmicutes	g__uncultured	C+AB	0.26	0.0026	0.0087
1648581785	p__Firmicutes	g__uncultured	CDC	0.1	0.001	0.003
1648581785	p__Firmicutes	g__uncultured	CDI	0.01	0.0001	0.0005
1648581787	p__Bacteroidetes	g__Bacteroides	C-AB	0.08	0.0008	0.0015
1648581787	p__Bacteroidetes	g__Bacteroides	C+AB	0.29	0.0029	0.007
1648581787	p__Bacteroidetes	g__Bacteroides	CDC	0.09	0.0009	0.0029
1648581787	p__Bacteroidetes	g__Bacteroides	CDI	0.63	0.0063	0.0241
1648581792	p__Actinobacteria	g__Collinsella	C-AB	0.41	0.0041	0.0046
1648581792	p__Actinobacteria	g__Collinsella	C+AB	0.43	0.0043	0.0068
1648581792	p__Actinobacteria	g__Collinsella	CDC	0.39	0.0039	0.0123
1648581792	p__Actinobacteria	g__Collinsella	CDI	0.01	0.0001	0.0003
1648581796	p__Firmicutes	g__Coprococcus_1	C-AB	0.53	0.0053	0.0063
1648581796	p__Firmicutes	g__Coprococcus_1	C+AB	0.29	0.0029	0.0037
1648581796	p__Firmicutes	g__Coprococcus_1	CDC	0.23	0.0023	0.0047
1648581796	p__Firmicutes	g__Coprococcus_1	CDI	0.04	0.0004	0.0018
1648581801	p__Firmicutes	g__[Ruminococcus]_gnavus_group	C-AB	0	0	0.0001
1648581801	p__Firmicutes	g__[Ruminococcus]_gnavus_group	C+AB	0.1	0.001	0.0019
1648581801	p__Firmicutes	g__[Ruminococcus]_gnavus_group	CDC	0.15	0.0015	0.0034
1648581801	p__Firmicutes	g__[Ruminococcus]_gnavus_group	CDI	0.25	0.0025	0.0034
1648581841	p__Firmicutes	g__Flavonifactor	C-AB	0.02	0.0002	0.0004
1648581841	p__Firmicutes	g__Flavonifactor	C+AB	0.06	0.0006	0.0009
1648581841	p__Firmicutes	g__Flavonifactor	CDC	0.22	0.0022	0.0038
1648581841	p__Firmicutes	g__Flavonifactor	CDI	0.46	0.0046	0.0068
1648581866	p__Firmicutes	g__Blautia	C-AB	0.31	0.0031	0.0023
1648581866	p__Firmicutes	g__Blautia	C+AB	0.26	0.0026	0.0025
1648581866	p__Firmicutes	g__Blautia	CDC	0.27	0.0027	0.0034
1648581866	p__Firmicutes	g__Blautia	CDI	0.07	0.0007	0.0016
1648581879	p__Euryarchaeota	g__Methanobrevibacter	C-AB	1.13	0.0113	0.0205
1648581879	p__Euryarchaeota	g__Methanobrevibacter	C+AB	1.56	0.0156	0.0394
1648581879	p__Euryarchaeota	g__Methanobrevibacter	CDC	0.72	0.0072	0.0165
1648581879	p__Euryarchaeota	g__Methanobrevibacter	CDI	0.08	0.0008	0.0027
1648581880	p__Firmicutes	g__Blautia	C-AB	0.32	0.0032	0.0028

1648581880	p__Firmicutes	g__Blautia	C+AB	0.26	0.0026	0.0028
1648581880	p__Firmicutes	g__Blautia	CDC	0.28	0.0028	0.0036
1648581880	p__Firmicutes	g__Blautia	CDI	0.08	0.0008	0.0017
1648581900	p__Firmicutes	g__Blautia	C-AB	0.15	0.0015	0.0013
1648581900	p__Firmicutes	g__Blautia	C+AB	0.16	0.0016	0.0021
1648581900	p__Firmicutes	g__Blautia	CDC	0.17	0.0017	0.0022
1648581900	p__Firmicutes	g__Blautia	CDI	0.05	0.0005	0.0012
164858191	p__Firmicutes	g__Blautia	C-AB	5.94	0.0594	0.0416
164858191	p__Firmicutes	g__Blautia	C+AB	4.77	0.0477	0.081
164858191	p__Firmicutes	g__Blautia	CDC	3	0.03	0.0652
164858191	p__Firmicutes	g__Blautia	CDI	0.51	0.0051	0.0161
1648581942	p__Firmicutes	g__Fusicatenibacter	C-AB	2.75	0.0275	0.0407
1648581942	p__Firmicutes	g__Fusicatenibacter	C+AB	2.26	0.0226	0.0405
1648581942	p__Firmicutes	g__Fusicatenibacter	CDC	0.42	0.0042	0.0089
1648581942	p__Firmicutes	g__Fusicatenibacter	CDI	0.63	0.0063	0.0273
1648581980	p__Firmicutes	g__Blautia	C-AB	0.17	0.0017	0.0016
1648581980	p__Firmicutes	g__Blautia	C+AB	0.17	0.0017	0.0019
1648581980	p__Firmicutes	g__Blautia	CDC	0.17	0.0017	0.0023
1648581980	p__Firmicutes	g__Blautia	CDI	0.04	0.0004	0.001
1648581991	p__Firmicutes	g__Subdoligranulum	C-AB	2.28	0.0228	0.0321
1648581991	p__Firmicutes	g__Subdoligranulum	C+AB	1.15	0.0115	0.0152
1648581991	p__Firmicutes	g__Subdoligranulum	CDC	2.49	0.0249	0.0533
1648581991	p__Firmicutes	g__Subdoligranulum	CDI	0.22	0.0022	0.0064
1648581996	p__Firmicutes	g__Blautia	C-AB	0.18	0.0018	0.0016
1648581996	p__Firmicutes	g__Blautia	C+AB	0.18	0.0018	0.0021
1648581996	p__Firmicutes	g__Blautia	CDC	0.19	0.0019	0.0024
1648581996	p__Firmicutes	g__Blautia	CDI	0.05	0.0005	0.0011
1648582	p__Firmicutes	g__Sellimonas	C-AB	0.01	0.0001	0.0002
1648582	p__Firmicutes	g__Sellimonas	C+AB	1.34	0.0134	0.0368
1648582	p__Firmicutes	g__Sellimonas	CDC	0.48	0.0048	0.0093
1648582	p__Firmicutes	g__Sellimonas	CDI	0.61	0.0061	0.0139
164858201	p__Firmicutes	g__Enterococcus	C-AB	0	0	0
164858201	p__Firmicutes	g__Enterococcus	C+AB	0.07	0.0007	0.0016
164858201	p__Firmicutes	g__Enterococcus	CDC	0.12	0.0012	0.0028
164858201	p__Firmicutes	g__Enterococcus	CDI	0.17	0.0017	0.0027
1648582144	p__Firmicutes	g__Ruminococcus_2	C-AB	1.6	0.016	0.0247
1648582144	p__Firmicutes	g__Ruminococcus_2	C+AB	2.24	0.0224	0.0493
1648582144	p__Firmicutes	g__Ruminococcus_2	CDC	1.07	0.0107	0.029
1648582144	p__Firmicutes	g__Ruminococcus_2	CDI	0.13	0.0013	0.0077
164858216	p__Firmicutes	g__Blautia	C-AB	0.67	0.0067	0.0043
164858216	p__Firmicutes	g__Blautia	C+AB	0.62	0.0062	0.0053
164858216	p__Firmicutes	g__Blautia	CDC	0.65	0.0065	0.0076
164858216	p__Firmicutes	g__Blautia	CDI	0.17	0.0017	0.0034
1648582163	p__Bacteroidetes	g__Bacteroides	C-AB	0.69	0.0069	0.0147
1648582163	p__Bacteroidetes	g__Bacteroides	C+AB	0.36	0.0036	0.0105
1648582163	p__Bacteroidetes	g__Bacteroides	CDC	0.85	0.0085	0.0417
1648582163	p__Bacteroidetes	g__Bacteroides	CDI	1.22	0.0122	0.026

1648582252	p__Firmicutes	g__Romboutsia	C-AB	1.4	0.014	0.0166
1648582252	p__Firmicutes	g__Romboutsia	C+AB	2.64	0.0264	0.0576
1648582252	p__Firmicutes	g__Romboutsia	CDC	1.61	0.0161	0.0395
1648582252	p__Firmicutes	g__Romboutsia	CDI	0.99	0.0099	0.0601
1648582328	p__Firmicutes	g__uncultured	C-AB	0.01	0.0001	0.0005
1648582328	p__Firmicutes	g__uncultured	C+AB	0.25	0.0025	0.007
1648582328	p__Firmicutes	g__uncultured	CDC	1.15	0.0115	0.0327
1648582328	p__Firmicutes	g__uncultured	CDI	0.4	0.004	0.0107
164858244	p__Firmicutes	g__Erysipelotrichaceae_UCG-003	C-AB	0.79	0.0079	0.0096
164858244	p__Firmicutes	g__Erysipelotrichaceae_UCG-003	C+AB	0.99	0.0099	0.03
164858244	p__Firmicutes	g__Erysipelotrichaceae_UCG-003	CDC	0.31	0.0031	0.0066
164858244	p__Firmicutes	g__Erysipelotrichaceae_UCG-003	CDI	0	0	0.0003
1648582527	p__Actinobacteria	g__Bifidobacterium	C-AB	3.35	0.0335	0.0357
1648582527	p__Actinobacteria	g__Bifidobacterium	C+AB	2.39	0.0239	0.0548
1648582527	p__Actinobacteria	g__Bifidobacterium	CDC	1.08	0.0108	0.0487
1648582527	p__Actinobacteria	g__Bifidobacterium	CDI	0.01	0.0001	0.0002
164858257	p__Firmicutes	g__Enterococcus	C-AB	0	0	0
164858257	p__Firmicutes	g__Enterococcus	C+AB	0.1	0.001	0.002
164858257	p__Firmicutes	g__Enterococcus	CDC	0.13	0.0013	0.0031
164858257	p__Firmicutes	g__Enterococcus	CDI	0.17	0.0017	0.0028
1648582610	p__Firmicutes	g__Ruminococcus_2	C-AB	2.36	0.0236	0.0679
1648582610	p__Firmicutes	g__Ruminococcus_2	C+AB	0.34	0.0034	0.0122
1648582610	p__Firmicutes	g__Ruminococcus_2	CDC	0.57	0.0057	0.0182
1648582610	p__Firmicutes	g__Ruminococcus_2	CDI	0.05	0.0005	0.0022
1648582724	p__Actinobacteria	g__Bifidobacterium	C-AB	0.47	0.0047	0.0058
1648582724	p__Actinobacteria	g__Bifidobacterium	C+AB	1.42	0.0142	0.0335
1648582724	p__Actinobacteria	g__Bifidobacterium	CDC	1.26	0.0126	0.0269
1648582724	p__Actinobacteria	g__Bifidobacterium	CDI	0.6	0.006	0.0249
164858286	p__Firmicutes	g__Enterococcus	C-AB	0	0	0.0001
164858286	p__Firmicutes	g__Enterococcus	C+AB	0.12	0.0012	0.0026
164858286	p__Firmicutes	g__Enterococcus	CDC	0.17	0.0017	0.0039
164858286	p__Firmicutes	g__Enterococcus	CDI	0.25	0.0025	0.0039
164858293	p__Firmicutes	g__Enterococcus	C-AB	0	0	0
164858293	p__Firmicutes	g__Enterococcus	C+AB	0.08	0.0008	0.0017
164858293	p__Firmicutes	g__Enterococcus	CDC	0.12	0.0012	0.0027
164858293	p__Firmicutes	g__Enterococcus	CDI	0.16	0.0016	0.0026
1648582959	p__Firmicutes	g__Enterococcus	C-AB	0	0	0
1648582959	p__Firmicutes	g__Enterococcus	C+AB	0.03	0.0003	0.0005
1648582959	p__Firmicutes	g__Enterococcus	CDC	0.46	0.0046	0.021
1648582959	p__Firmicutes	g__Enterococcus	CDI	0.14	0.0014	0.0034
164858296	p__Firmicutes	g__Blautia	C-AB	0.22	0.0022	0.0018
164858296	p__Firmicutes	g__Blautia	C+AB	0.25	0.0025	0.0026
164858296	p__Firmicutes	g__Blautia	CDC	0.21	0.0021	0.0027
164858296	p__Firmicutes	g__Blautia	CDI	0.05	0.0005	0.0012
1648582992	p__Firmicutes	g__Blautia	C-AB	0.41	0.0041	0.0056
1648582992	p__Firmicutes	g__Blautia	C+AB	0.16	0.0016	0.0029
1648582992	p__Firmicutes	g__Blautia	CDC	0.18	0.0018	0.0063

1648582992	p__Firmicutes	g__Blautia	CDI	0.02	0.0002	0.0007
164858332	p__Firmicutes	g__[Clostridium]_innocuum_group	C-AB	0.04	0.0004	0.0007
164858332	p__Firmicutes	g__[Clostridium]_innocuum_group	C+AB	0.94	0.0094	0.0182
164858332	p__Firmicutes	g__[Clostridium]_innocuum_group	CDC	0.98	0.0098	0.0183
164858332	p__Firmicutes	g__[Clostridium]_innocuum_group	CDI	2.51	0.0251	0.0846
164858377	p__Firmicutes	g__Blautia	C-AB	0.3	0.003	0.0025
164858377	p__Firmicutes	g__Blautia	C+AB	0.29	0.0029	0.0033
164858377	p__Firmicutes	g__Blautia	CDC	0.29	0.0029	0.0036
164858377	p__Firmicutes	g__Blautia	CDI	0.08	0.0008	0.0017
164858382	p__Firmicutes	g__Lachnoclostridium	C-AB	0	0	0.0001
164858382	p__Firmicutes	g__Lachnoclostridium	C+AB	0.03	0.0003	0.0004
164858382	p__Firmicutes	g__Lachnoclostridium	CDC	0.1	0.001	0.0027
164858382	p__Firmicutes	g__Lachnoclostridium	CDI	0.25	0.0025	0.0053
1648584	p__Bacteroidetes	g__Bacteroides	C-AB	2.35	0.0235	0.0471
1648584	p__Bacteroidetes	g__Bacteroides	C+AB	2.34	0.0234	0.0451
1648584	p__Bacteroidetes	g__Bacteroides	CDC	0.56	0.0056	0.0158
1648584	p__Bacteroidetes	g__Bacteroides	CDI	5.29	0.0529	0.101
1648584047	p__Firmicutes	g__Lactobacillus	C-AB	0.04	0.0004	0.0012
1648584047	p__Firmicutes	g__Lactobacillus	C+AB	0.07	0.0007	0.0019
1648584047	p__Firmicutes	g__Lactobacillus	CDC	0.06	0.0006	0.0021
1648584047	p__Firmicutes	g__Lactobacillus	CDI	0.55	0.0055	0.0195
164858467	p__Firmicutes	g__Veillonella	C-AB	0.03	0.0003	0.0003
164858467	p__Firmicutes	g__Veillonella	C+AB	0.05	0.0005	0.001
164858467	p__Firmicutes	g__Veillonella	CDC	0.36	0.0036	0.0198
164858467	p__Firmicutes	g__Veillonella	CDI	0.66	0.0066	0.0182
164858499	p__Firmicutes	g__Erysipelatoclostridium	C-AB	0.11	0.0011	0.0025
164858499	p__Firmicutes	g__Erysipelatoclostridium	C+AB	0.51	0.0051	0.0077
164858499	p__Firmicutes	g__Erysipelatoclostridium	CDC	1.17	0.0117	0.0485
164858499	p__Firmicutes	g__Erysipelatoclostridium	CDI	1.06	0.0106	0.0158
1648585	p__Firmicutes	g__[Ruminococcus]_gauvreauii_group	C-AB	1.21	0.0121	0.019
1648585	p__Firmicutes	g__[Ruminococcus]_gauvreauii_group	C+AB	0.69	0.0069	0.0164
1648585	p__Firmicutes	g__[Ruminococcus]_gauvreauii_group	CDC	0.13	0.0013	0.0039
1648585	p__Firmicutes	g__[Ruminococcus]_gauvreauii_group	CDI	0.14	0.0014	0.0079
164858516	p__Firmicutes	g__Lactobacillus	C-AB	0.03	0.0003	0.0011
164858516	p__Firmicutes	g__Lactobacillus	C+AB	0.8	0.008	0.0229
164858516	p__Firmicutes	g__Lactobacillus	CDC	0.62	0.0062	0.031
164858516	p__Firmicutes	g__Lactobacillus	CDI	0.43	0.0043	0.015
1648585657	p__Firmicutes	g__Enterococcus	C-AB	0	0	0
1648585657	p__Firmicutes	g__Enterococcus	C+AB	0.08	0.0008	0.0031
1648585657	p__Firmicutes	g__Enterococcus	CDC	0.09	0.0009	0.0035
1648585657	p__Firmicutes	g__Enterococcus	CDI	0.31	0.0031	0.0081
16485877	p__Firmicutes	g__Blautia	C-AB	0	0	0.0001
16485877	p__Firmicutes	g__Blautia	C+AB	0.35	0.0035	0.0075
16485877	p__Firmicutes	g__Blautia	CDC	0.59	0.0059	0.0113
16485877	p__Firmicutes	g__Blautia	CDI	1.13	0.0113	0.0405
164858777	p__Firmicutes	g__Enterococcus	C-AB	0	0	0
164858777	p__Firmicutes	g__Enterococcus	C+AB	0.06	0.0006	0.0015

164858777	p__Firmicutes	g__Enterococcus	CDC	0.12	0.0012	0.0027
164858777	p__Firmicutes	g__Enterococcus	CDI	0.15	0.0015	0.0025
16485879	p__Firmicutes	g__Streptococcus	C-AB	4.32	0.0432	0.0741
16485879	p__Firmicutes	g__Streptococcus	C+AB	4.28	0.0428	0.0735
16485879	p__Firmicutes	g__Streptococcus	CDC	3.87	0.0387	0.0757
16485879	p__Firmicutes	g__Streptococcus	CDI	2.65	0.0265	0.0877
1648588	p__Firmicutes	g__Enterococcus	C-AB	0.17	0.0017	0.0025
1648588	p__Firmicutes	g__Enterococcus	C+AB	3.38	0.0338	0.0681
1648588	p__Firmicutes	g__Enterococcus	CDC	5.92	0.0592	0.1396
1648588	p__Firmicutes	g__Enterococcus	CDI	7.75	0.0775	0.1347
1648588051	p__Firmicutes	g__[Ruminococcus]_torques_group	C-AB	1.21	0.0121	0.014
1648588051	p__Firmicutes	g__[Ruminococcus]_torques_group	C+AB	0.49	0.0049	0.011
1648588051	p__Firmicutes	g__[Ruminococcus]_torques_group	CDC	0.72	0.0072	0.0251
1648588051	p__Firmicutes	g__[Ruminococcus]_torques_group	CDI	0.28	0.0028	0.0133
1648588054	p__Firmicutes	g__Lachnospiraceae_ND3007_group	C-AB	0.62	0.0062	0.0105
1648588054	p__Firmicutes	g__Lachnospiraceae_ND3007_group	C+AB	0.16	0.0016	0.003
1648588054	p__Firmicutes	g__Lachnospiraceae_ND3007_group	CDC	0.2	0.002	0.0045
1648588054	p__Firmicutes	g__Lachnospiraceae_ND3007_group	CDI	0.02	0.0002	0.0007
164858888	p__Firmicutes	g__Blautia	C-AB	0.14	0.0014	0.0015
164858888	p__Firmicutes	g__Blautia	C+AB	0.16	0.0016	0.0017
164858888	p__Firmicutes	g__Blautia	CDC	0.16	0.0016	0.0022
164858888	p__Firmicutes	g__Blautia	CDI	0.04	0.0004	0.0008
164858927	p__Firmicutes	g__Blautia	C-AB	0.17	0.0017	0.0015
164858927	p__Firmicutes	g__Blautia	C+AB	0.15	0.0015	0.0017
164858927	p__Firmicutes	g__Blautia	CDC	0.19	0.0019	0.0026
164858927	p__Firmicutes	g__Blautia	CDI	0.05	0.0005	0.001
164858931	p__Firmicutes	g__[Ruminococcus]_gnavus_group	C-AB	0.08	0.0008	0.001
164858931	p__Firmicutes	g__[Ruminococcus]_gnavus_group	C+AB	1.5	0.015	0.0306
164858931	p__Firmicutes	g__[Ruminococcus]_gnavus_group	CDC	2.07	0.0207	0.0532
164858931	p__Firmicutes	g__[Ruminococcus]_gnavus_group	CDI	3.49	0.0349	0.05
