# Supplementary figures and images for: The Bacterial Gut Microbiota of Adult Patients Infected, Colonized or Noncolonized by Clostridioides difficile
Source: Microorganisms. 2020 May 6;8(5):677. doi: 10.3390/microorganisms8050677 (PMC7284656; doi:10.3390/microorganisms8050677)

**A Ribotypes among CDC patients**

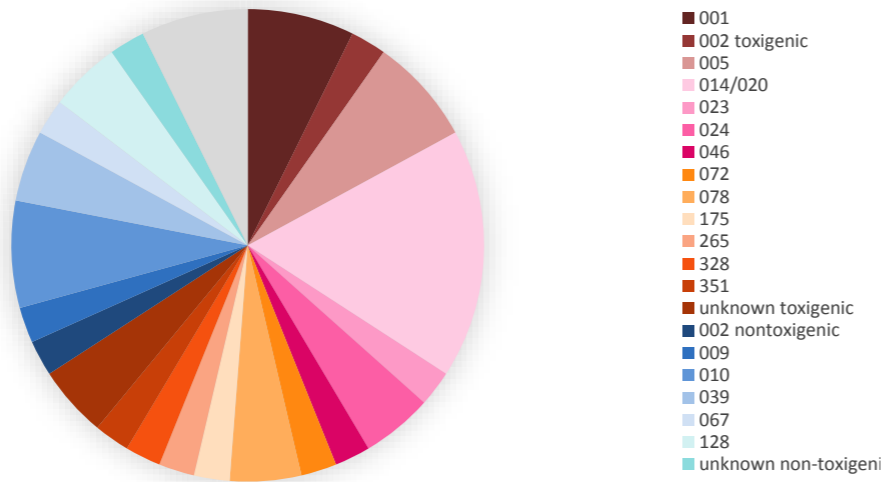

**B Ribotypes among CDI patients**

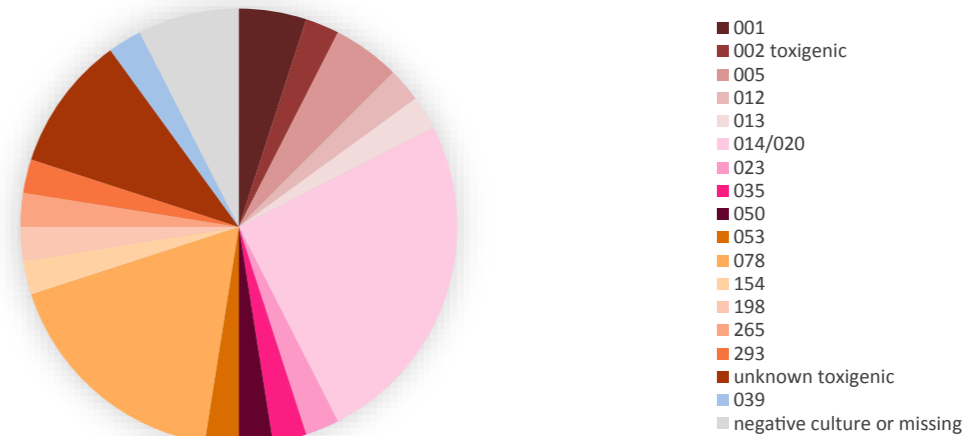

Supplement: Supplementary file 1 [file microorganisms-08-00677-s001.zip › microorganisms-795617-supplementary-proofreading/Figure_S2_Pie_Charts_Distribution.pdf]
